# Unravelling Marine Benthic Functioning Shifts Under Ocean Acidification

**DOI:** 10.1111/ele.70376

**Published:** 2026-04-03

**Authors:** J. Carlot, S. Comeau, A. Chiarore, A. Mirasole, S. Alliouane, F. Micheli, C. L. Hurd, J.‐P. Gattuso, N. Teixidó

**Affiliations:** ^1^ Laboratoire d'Océanographie de Villefranche Sorbonne Université Villefranche‐sur‐mer France; ^2^ Institute of Marine Sciences‐CSIC (ICM‐CSIC) Barcelona Spain; ^3^ Stazione Zoologica Anton Dohrn, National Institute of Marine Biology, Ecology and Biotechnology Ischia Marine Center Ischia Italy; ^4^ Oceans Department, Hopkins Marine Station and Stanford Center for Ocean Solutions, Stanford Doerr School of Sustainability Stanford University Pacific Grove California USA; ^5^ Ecology and Biodiversity Institute for Marine and Antarctic Studies (IMAS) Battery Point Tasmania Australia; ^6^ Institute for Sustainable Development and International Relations Paris France

**Keywords:** benthic communities, CO_2_ vents, ecosystem functioning, ocean acidification, shifts

## Abstract

Ocean acidification (OA) driven by increasing atmospheric CO_2_ is altering marine biodiversity. However, impacts of OA on ecosystem functioning at the community level, including calcification, primary production and nutrient uptake, remain largely unknown. Here, we conducted community transplant experiments at natural CO_2_ vents to assess how declining pH affects marine community species composition, biomass, and key ecosystem processes over time. Our results indicate that community shifts caused by declining pH lead to decreased biomass and calcification rates, while photosynthesis and nutrient uptake rates increased. By leveraging OA field model systems and in situ measurements of ecosystem functioning, this study provides critical insights into how OA‐induced biodiversity loss reshapes the structure and functioning of temperate marine coastal ecosystems.

## Graphical abstract

Ocean acidification is reshaping temperate shallow marine benthic communities and the way they function. Using community transplants at natural CO_2_ vents, we show that biodiversity loss under declining pH reduces biomass and calcification, while enhancing photosynthesis and nutrient uptake. These functional shifts reveal how ocean acidification fundamentally reorganizes coastal ecosystem functioning.

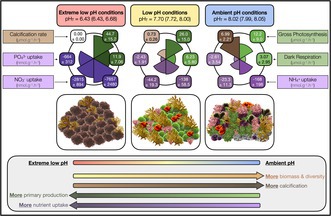

## 1 Introduction

Ocean Acidification (OA), caused by the ocean's uptake of approximately 25% of anthropogenic CO_2_ emissions (Gattuso and Hansson 2011), is expected to pose a major threat to marine ecosystems because it reduces the capacity of calcifying organisms such as molluscs (Rodolfo‐Metalpa et al. 2011) and corals (Mollica et al. 2018) to form their shells or skeletons. Most studies investigating the effects of OA at the ecosystem level have already revealed severe declines in biodiversity and habitat complexity (Peña et al. 2021; Agostini et al. 2018; Kroeker et al. 2013; Hall‐Spencer et al. 2008). However, our understanding of how biodiversity loss affects ecosystem functioning (EF) under OA remains limited, particularly for key functions such as calcification, primary production and nutrient uptake.

Generally, higher biodiversity enhances the ecological resilience and stability of the ecosystem (Wang and Loreau 2016). As OA intensifies, there is growing concern that marine ecosystems may become increasingly simplified, with the loss of complex habitat‐forming species including calcifying algae, corals or oysters to the benefit of simpler algal‐dominated assemblages such as low‐lying, centimetre‐tall algae, hereafter referred to as turf algae (Agostini et al. 2021). These compositional shifts can decrease functional redundancy, potentially diminishing ecosystem productivity and stability (Biggs et al. 2020). EF, commonly defined as the fluxes of energy or material within an ecosystem, is strongly influenced by species composition and diversity (Bellwood et al. 2019; Brandl et al. 2023). While many studies suggest that biodiversity loss can lead to EF declines (Mori et al. 2018; Van Der Plas 2019), EF responses are likely more complex and might be enhanced or compensated in some cases (Armitage et al. 2024; Micheli and Halpern 2005). For example, the additional CO_2_ in the ocean, which causes OA, might favour photosynthesis in calcifying primary producers but impair their calcification rates (Gao et al. 2019). To date, few studies have quantified the impacts of OA on EF and the possible shifts between different functioning rates in the field and at the community level (Cornwall et al. 2024), due to logistical challenges in studying intact ecosystems under natural conditions while maintaining ecosystem integrity.

To overcome these challenges, submarine volcanic CO_2_ vents offer an invaluable opportunity to investigate shifts in community diversity, structure, and EF in response to OA (Hall‐Spencer et al. 2008). One of these is the CO_2_ vents of Ischia (Italy), which have been active for decades (Foo et al. 2018) and present a natural pH gradient, providing pH conditions ranging from low‐emission (SSP1‐2.6) to high‐emission (SSP5‐8.5) CO_2_ scenarios (−0.16 to −0.44 pH_T_, pH on total scale) and extreme low pH zones to explore OA impacts further (Teixido et al. 2018). By studying these high‐CO_2_ environments and their communities, it becomes possible to predict and quantify marine EF responses to global environmental change, thereby improving our understanding of the links between biodiversity and EF in complex marine ecosystems.

Here, we extend previous trait‐based work under OA (Teixido et al. 2024) by moving from species‐level trait patterns to community‐scale measurements of EF in order to reveal how changes in species composition and biomass shape ecosystem‐level responses to acidification. We deployed settlement tiles in ambient pH conditions and transplanted them across distinct pH zones to quantify how these compositional changes translate into shifts in key ecosystem functions—calcification, gross photosynthesis, dark respiration and nutrient uptake. Unlike trait‐based approaches focusing on presence–absence, our community‐level measurements capture functional shifts arising from species reorganization and changes in biomass. We show that OA simplifies communities through the loss of calcifying taxa and the proliferation of fleshy and turf algae, generating directional shifts in EF across three communities. By integrating compositional dynamics with direct functional measurements, our study provides new insights into how OA‐driven biodiversity loss can restructure temperate reef functioning, shedding light on long‐term ecological dynamics in a changing ocean.

## 2 Methods

### 2.1 Study Site

Volcanic CO_2_ vents are at 0.5–3 m water depth on the south and north sides of Castello Aragonese islet, Ischia, Italy, adjacent to sloping rocky reefs, and create a natural pH gradient (Hall‐Spencer et al. 2008). We followed the three pH zones corresponding to an extreme low pH zone (high venting activity); a low pH zone (moderate venting activity); and an ambient pH zone (non‐visible vent activity) on the south side (Teixido et al. 2018). Each zone spans ~20 m and is separated by 20–25 m. Ambient conditions reflect current average pH, low pH simulates future scenarios under SSP1‐2.6 to SSP5‐8.5 (−0.16 to −0.44 pH), and extreme low pH provides an extreme scenario to test responses to OA.

### 2.2 pH_T_ Time Series, pH_T_ Variability and Carbonate Chemistry Across pH Zones

To characterize the pH, a SeaFET sensor (SeaBird) and two Sami sensors (Sunburst) were deployed in the three pH zones (extreme low, low, ambient) at the same depths as the settlement tile transplants (0.5–3 m; Figure S1). Sensors recorded every 15 min from May 8–June 26 (spring) and September 11–20, 2023 (early fall). The SeaFET was calibrated in ambient seawater with spectrophotometric pH determination, showing a mean offset of ±0.0001 units (*n* = 12; Dickson et al. 2007). Sami sensors were verified against TRIS buffers (Batches T38–T39; Dickson et al. 2007), with deviations from nominal values ranging from −0.0160 to −0.001. Discrete water samples for total alkalinity (A_T_) were collected concurrently and measured using the open‐cell potentiometric method (Dickson et al. 2007). The HCl (0.1 M) titrant was calibrated against Certified Reference Materials (Batch #184; Dickson et al. 2007), with mean differences from nominal values < 5 μmol.kg^−1^ (Riebesell et al. 2011). A_T_, pH_T_, and in situ temperature, salinity and depth were then used to calculate the remaining carbonate system parameters with the R package *seacarb* v3.3.3 (Gattuso et al. 2019).

### 2.3 Transplantation Experiments Using Settlement Tiles

Two experiments using volcanic rock recruitment tiles were conducted to investigate ecological functions (calcification, gross photosynthesis and nutrients uptake) shifts across pH zones. In June 2013, 18 recruitment tiles (15 × 15 × 1 cm) were deployed at 0.5–1.5 m depth across all three pH zones (*n* = 6 per zone) and monitored at two time points (T_*n*1_, T_*n*2_; referred to as historic tiles). In November 2019, 18 recruitment tiles (15 × 15 × 2 cm) were deployed in the ambient zone and allowed to recolonize for 3.5 years (referred to as transplanted tiles). In May 2023, transplanted tiles were randomly placed across extreme low, low, and ambient pH zones (*n* = 6 per zone, 0.5–1.5 m depth; T_0_) and we performed incubation experiments on individual tiles to measure ecological responses at T_0_, 7 days (T_1_), 1 month (T_2_), and 4 months (T_3_) (Figure S2). Historic tiles at T_*n*1_ and T_*n*2_ were assessed at the same time as T_2_ and T_3_ of the transplanted tiles experiment.

### 2.4 Net Photosynthesis and Dark Respiration Rate Measurements

Net photosynthesis and dark respiration were measured by incubating entire tiles in custom‐built 3.7 L chambers equipped with 12 V submersible pumps (maximum flow rate of 280 L h^−1^) to ensure continuous water circulation during the incubations. Chambers were sealed with a watertight lid fitted with a tap to prevent mixing with ambient seawater. Four transparent chambers (referred to as light chambers) allowed light penetration for net photosynthesis, while four black chambers (referred to as dark chambers) blocked light, to measure dark respiration. In each experimental set, three chambers contained individual tiles and one served as a control for background metabolic rates. Tiles in light chambers were incubated for 75 min to avoid O_2_ supersaturation (> 150%), which can inhibit photosynthesis and alter pH (McMinn et al. 2005). Dark incubations lasted 60 min to maintain O_2_ > 80% saturation, preventing stress in benthic fauna (Kolb 2018). Oxygen was recorded every minute with factory‐calibrated Minidots sensors (PME, USA; https://www.pme.com/products/minidot). To ensure measurement accuracy, sensors were weekly calibrated at 100% O_2_ saturation by immersion in seawater bubbled with atmospheric air. After each set, tiles were swapped between light and dark chambers following a 30 min acclimation. Gross photosynthesis was calculated as net photosynthesis minus dark respiration, expressed as μmol O_2_ h^−1^ per tile (Figure S3).

### 2.5 Nutrient Uptake and Calcification Rate Measurements

For each incubation, water samples were collected at the start and end to measure nutrient uptake and calcification. Duplicate 20 mL samples for nutrients were stored in high‐density polyethylene bottles at −20°C, and 250 mL samples for total alkalinity (A_T_) in borosilicate bottles at 4°C (max. 3 days). Prior to A_T_ analysis, samples were equilibrated to room temperature (23°C) for 1 h. Calcification was calculated from the change in A_T_ (∆A_T_) during incubations, assuming a 2:1 stoichiometry between A_T_ decrease and CaCO₃ precipitation, corrected for seawater density (1.025 kg L^−1^) and chamber volume (3.7 L), and expressed as μmol CaCO₃ h^−1^ per tile. A_T_ was determined in duplicate or triplicate following SOP3a (Dickson et al. 2007) (Figure S3). Ammonium (NH₄^+^), nitrate (NO₃^−^), and phosphate (PO₄^3−^) concentrations were quantified using a Flowsys III Continuous Flow Analyser (Systea), with detection limits of 0.05 mmol m^−3^ (NH₄^+^) and 0.01 mmol m^−3^ (NO₃^−^, PO₄^3−^). Nutrient uptake was calculated as the difference between final and initial concentrations, corrected by controls and standardized over time. Fluxes were reported in μmol h^−1^ per tile.

### 2.6 Adjustments for Photosynthetically Active Radiation

Relationships between metabolic rates and irradiance were assessed for each pH zone and experimental time point using three tiles per zone (*n* = 9). Irradiance during incubations was logged every 5 min with two PAR sensors (Odyssey Xtreem), cross‐calibrated against a LI‐COR quantum sensor (Li1400). Net photosynthesis was measured during 1 h incubations conducted every 2 h from 06:00 to 22:00 GMT+1 over three consecutive days, with mean PAR calculated for each incubation. Data were fitted with the equation *y* = *ɑ*(1—*β*.e^γx^) (Aalderink and Jovin 1997; *R* ^2^ = 0.52–0.73), where ɑ is the maximum photosynthetic rate and β and γ are curvature and saturation constants (Figure S4). These relationships were then used to estimate maximum net photosynthetic rates at the maximum PAR recorded (600 μmol m^−2^ s^−1^) for transplanted (T_0_–T_3_) and historic (T_*n*1_–T_*n*2_) tiles.

### 2.7 Species Identification and Biomass Estimate

At each time point (T_0_–T_3_ for transplanted tiles; T_*n*1_–T_*n*2_ for historic tiles), benthic species and their cover were identified to assess community composition. Percent cover was quantified through underwater photographs complemented by visual census. For each photograph, a 25‐square grid (5 × 5 cm) was superimposed on both sides of the tile, and occupied squares were counted to calculate percent cover, expressed as relative percentages (Teixidó et al. 2024). In total, 61 taxa were identified to the lowest possible level (25 algal species, 1 algal turf group and 35 invertebrates; Figure S5). Based on dominant taxa on the front side, communities were classified as calcifying algae‐, mixed algae‐ or fleshy algae‐dominated. To provide quantitative support for this classification, we performed 3D NMDS and PERMANOVA analyses, which confirmed the presence of three statistically distinct community types (PERMANOVA: *F* = 1.698, *R* ^2^ = 0.343, *p* = 0.022; see Figure S6). To estimate biomass, in situ triplicate 5 × 5 cm samples of each species were collected, dried at 50°C for 48 h and weighed. Species‐specific dry weights were then applied to percent cover data to calculate total biomass for each tile across the four transplanted (T_0_–T_3_) and two historic (T_*n*1_–T_*n*2_) time steps (Denny and Benedetti‐Cecchi 2012).

### 2.8 Change in Biomass and Ecological Functions

To quantify the changes in biomass and ecological processes (calcification, gross photosynthesis, and nutrient (NH₄^+^, NO₃^−^, PO₄^3−^) uptake), we standardized values at each time point relative to the initial measurement (T_0_ for transplanted tiles, T_*n*1_ for historic tiles). Standardized biomass values were strictly positive, while process rates could be either positive or negative. We applied two Bayesian non‐linear models using Weibull and Gaussian distributions and models were specified with the following structure:

$$B∼\text{Weibull}\left(\mu_{B},\sigma_{B}\right)$$

$$F∼\text{Normal}\left(\mu_{F},\sigma_{F}\right)$$

$$\mu =\left(\beta +{\text{ζ}}_{\text{communities}}+{\text{ζ}}_{\mathrm{pH}}\right)\times \text{time}$$

$$\text{ζ}={\left(\mathrm{\Omega Z}\right)}_{s}$$

$$\mathrm{diag}\left(Z\right)={\text{𝜎}}_{\text{ζ}}$$

$$\begin{array}{c} \text{𝛽}∼\text{flat};\text{𝜎}∼\Gamma \left(0.01,0.01\right);{\text{𝛿}}_{s}∼\text{Student}\left(3,0,2.5\right);\\ \Omega ∼\mathrm{LKJ}\left(1\right);{\text{𝜎}}_{\text{ζ}}∼\Gamma \left(0.01,0.01\right) \end{array}$$

where *B* and *F* represent the standardized biomass and process rates respectively, and time represents the experiment duration in days. *β* denotes the overall scaling slope for communities and pH. The models include communities (calcifying algae‐dominated, mixed algae‐dominated and fleshy algae‐dominated communities), a vector of *n*_1_ = 3 levels of communities observed and pH, a vector of *n*_2_ = 3 levels of pH sites. These vectors construct a hierarchical matrix 𝜁 with *n*_1_ × *n*_2_ rows and two columns, representing both community‐level and pH‐level additive deviations from β. In the models, Ω is the Cholesky factor of the correlation matrix among hierarchical effects, *Z* is a diagonal matrix with a vector of among‐communities and among‐pH standard deviations (𝜎_𝜁_) and 𝛿_s_ is an s‐by‐two matrix of standardized hierarchical effects. We considered an effect to be meaningful between two conditions when their respective 75% credible intervals did not overlap, indicating high certainty about the direction of the effect. Models were run with four chains, 10,000 iterations each, 2000 warm‐up steps, retaining 32,000 posterior draws. Convergence was checked via trace plots and *R*_hat_ (< 1.05; Gelman and Rubin 1992), with model fits of *R* ^2^ = 0.77 for biomass, 0.22–0.43 for ecological functions using the transplanted tiles and 0.38–0.63 for ecological functions using the historic tiles. All analyses were conducted in R 4.3.2 using the R package *brms* (Bürkner 2017).

## 3 Results

### 3.1 Change in Physiochemical Parameters Across Sites

**FIGURE 1:**
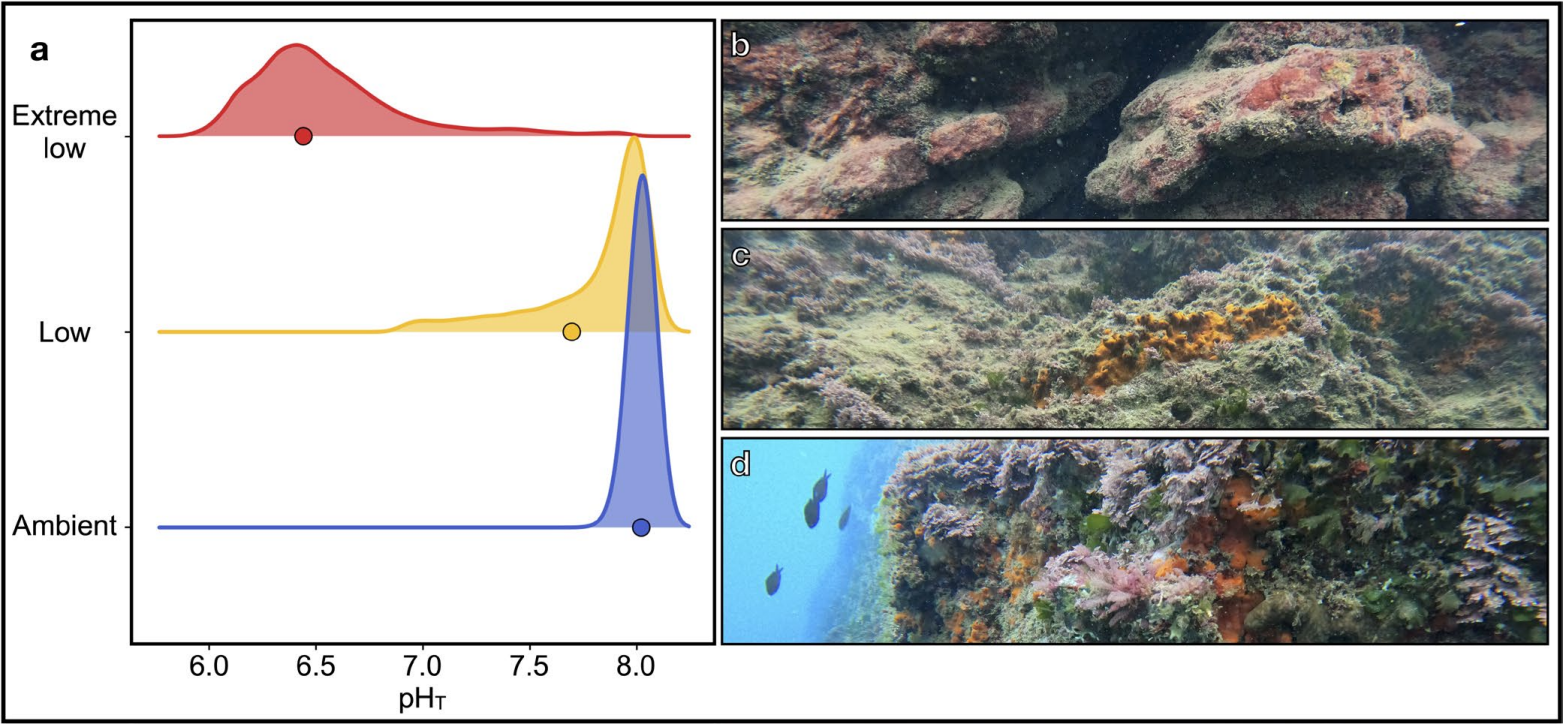
Distribution of pH_T_ measures in the extreme low, low and ambient pH zones. (a) Measurements were taken every 15 min in spring (08/05/23–26/06/23) and fall (11/09/23–20/09/23), totalling 17,475 measurements (*n* = 6342 ambient pH; *n* = 5587 low pH and *n* = 5546 extreme low pH zones). The solid dots represent the mean pH_T_. The mean carbonate chemistry in the ambient pH zone corresponds to current average conditions, whereas the low pH site is most comparable with values predicted for the year 2100 with a decrease in surface pH from −0.16 to −0.44 pH units under SSP1‐2.6 and SSP5‐8.5. The extreme low pH zone is used to represent more extreme scenarios. (b–d) Seascape at the three pH zones: (b) Extreme low pH zone characterized by the encrusting non‐calcareous perennial red algae *Hildenbrandia crouaniorum* and turf algae; (c) Low pH zone characterized by fleshy algae, including *Flabellia petiolata*, *Halopteris scoparia* and the encrusting sponge *Crambe crambe*; and (d) Ambient pH zone characterized by fleshy algae, including *Halopteris scoparia* and *Dictyota* sp. and a variety of calcifying algae such as *Ellisolandia elongata*.

The three transplantation sites differed sharply in seawater pH (Figure 1). Extreme low pH tiles experienced mean pH_T_ = 6.43 (25th–75th percentile: 6.43–6.68), low pH tiles pH_T_ = 7.70 (7.72–8.00), displaying skewed values due to localized venting, and ambient pH tiles pH_T_ = 8.02 (7.99–8.05). Saturation states of calcite (Ωc) and aragonite (Ωa) followed the same gradient (Table 1). Extreme low pH conditions were severely undersaturated (Ωc = 0.3 ± 0.5, Ωa = 0.2 ± 0.3), low pH remained above undersaturation but was substantially lower than ambient (Ωc = 3.7 ± 1.4, Ωa = 2.4 ± 0.9), while ambient conditions were highly supersaturated (Ωc = 5.4 ± 0.4, Ωa = 3.5 ± 0.3).

**TABLE 1:** Measured and derived seawater physiochemical parameters at the study sites for salinity (S), temperature (T), total alkalinity (A_T_), dissolved inorganic carbon (C_T_), pH_T_, *p*CO_2_, calcite (Ωc) and aragonite (Ωa) saturation.

Season	pH conditions	T (°C)	A_T_ (μmol kg^−1^)	C_T_ (μmol kg^−1^)	pH_T_	*p*CO_2_ (μatm)	Ω_c_	Ω_a_
Spring	Ambient pH	21.7 ^b^ ± 2.5 (18.9, 23.8) *n* = 5013	2662^a^ ± 14 (2655, 2658) *n* = 4	2306 ± 49 (2254, 2345) *n* = 5012	8.03^b^ (8.01, 8.06) *n* = 5012	463 ± 58 (421, 494) *n* = 5013	5.4 ± 0.4 (5.1, 5.7) *n* = 5012	3.5 ± 0.3 (3.3, 3.7) *n* = 5012
	Low pH	22.0^b^ ± 2.6 (19.1, 24.3) *n* = 4738	2656^a^ ± 2 (2655, 2658) *n* = 3	2438 ± 135 (2342, 2497) *n* = 4571	7.71^b^ (7.75, 8.00) *n* = 4738	1092 ± 1268 (494, 974) *n* = 738	3.7 ± 1.4 (2.9, 4.7) *n* = 4705	2.4 ± 0.9 (1.9, 3.0) *n* = 4571
	Extreme low pH	22.0^b^ ± 2.7 (19.1, 24.3) *n* = 4705	2612^a^ ± 7 (2608, 2617) *n* = 4	3285 ± 412 (3000, 3518) *n* = 4705	6.48^b^ (6.35, 6.73) *n* = 4705	20,802 ± 12,354 (11,756, 28,043) *n* = 4705	0.4 ± 0.6 (0.1, 0.3) *n* = 4705	0.2 ± 0.4 (0.1, 0.2) *n* = 4705
Fall	Ambient pH	24.9^b^ ± 0.2 (24.7, 25.1) *n* = 1329	2662^a^ ± 14 (2651, 2674) *n* = 4	2282 ± 22 (2266, 2293) *n* = 1329	7.97^b^ (7.96, 8.00) *n* = 1329	529 ± 56 (490, 552) *n* = 1329	5.3 ± 0.3 (5.1, 5.6) *n* = 1329	3.5 ± 0.2 (3.4, 3.7) *n* = 1329
	Low pH	25.0^b^ ± 0.3 (24.7, 25.2) *n* = 849	2656^a^ ± 2 (2655, 2658) *n* = 3	2390 ± 131 (2290, 2480) *n* = 810	7.61^b^ (7.56, 7.94) *n* = 849	1390 ± 1369 (571, 1556) *n* = 810	3.6 ± 1.5 (2.3, 4.9) *n* = 810	2.4 ± 1.0 (1.5, 3.3) *n* = 810
	Extreme low pH	24.9^b^ ± 0.4 (24.6, 25.2) *n* = 841	2612^a^ ± 7 (2608, 2616) *n* = 4	3638 ± 428 (3340, 3920) *n* = 841	6.23^b^ (6.13, 6.37) *n* = 841	37,188 ± 14,762 (26,950, 46,934) *n* = 841	0.2 ± 0.1 (0.1, 0.2) *n* = 841	0.1 ± 0.1 (0.1, 0.1) *n* = 841
Total	Ambient pH	22.4^b^ ± 2.6 (19.4, 24.8) *n* = 6342	2662^a^ ± 14 (2651, 2674) *n* = 8	2301 ± 45 (2260, 2340) *n* = 6341	8.02^b^ (7.99, 8.05) *n* = 6342	477 ± 64 (431, 509) *n* = 6342	5.4 ± 0.4 (5.1, 5.6) *n* = 6341	3.5 ± 0.3 (3.3, 3.7) *n* = 6341
	Low pH	22.5^b^ ± 2.7 (19.4, 24.7) *n* = 5587	2656^a^ ± 2 (2655, 2658) *n* = 6	2431 ± 136 (2330, 2495) *n* = 5381	7.69^b^ (7.72, 8.00) *n* = 5587	1137 ± 1288 (505, 1058) *n* = 5587	3.7 ± 1.4 (2.8, 4.7) *n* = 5381	2.4 ± 0.9 (1.8, 3.1) *n* = 5381
	Extreme low pH	22.5^b^ ± 2.7 (19.5, 24.7) *n* = 5546	2612^a^ ± 7 (2608, 2616) *n* = 8	3338 ± 433 (3030, 3595) *n* = 5546	6.43^a^ (6.43, 6.68) *n* = 5546	23,287 ± 14,037 (12,909, 31,404) *n* = 5546	0.3 ± 0.5 (0.1, 0.3) *n* = 5546	0.2 ± 0.3 (0.1, 0.2) *n* = 5546

*Note:* Values are means ± SD, with 25th and 75th percentiles in parenthesis. Calculated concentrations of C_T_, *p*CO_2_, Ω_c_ and Ω_a_ are shown.

^a^ Parameters measured from discrete water samples.

^b^ Parameters measured in situ.

### 3.2 Change in Species Composition and Biomass due to OA

Under ambient pH conditions, all communities exhibited relatively constant cover over time, except for the fleshy algal‐dominated communities, which showed a marked decrease (−17% ± 13%; mean ± SD) overall in brown macroalgae (Phylum Ochrophyta, Class Phaeophyceae) over time, and an increase (20% ± 12%) in red macroalgal (Phylum Rhodophyta) cover due to the seasonal appearance of the small‐calcifying red alga *Haliptilon virgatum*, which becomes highly abundant after summer (Table 2, Figure S5). Under low pH, different patterns were observed across the different community types over time, with a notable loss of bryozoans on both sides of the tiles (−12% ± 7%) and a marked reduction in almost all brown algae except *Dictyota* sp. (−5.9% ± 11.0%). These communities also showed a pronounced increase (28.5% ± 1.5%) in turf cover (front side) and mortality of dim‐light animals (11.1% ± 6.4%) (back side) over time. Under extreme low pH, the cover of almost all taxa declined (between −30.0% ± 2.8% and −1.9% ± 1.3%) over time on both sides of the tiles, leaving only a few low‐pH‐tolerant species (e.g., the macroalgae *Hildenbrandia crouaniorum* and *Dictyota* sp.).

**TABLE 2:** Taxonomic groups, functional roles (calcifier, primary producer, filter‐feeder), and temporal changes in percent cover of key benthic taxa across extreme low, low and ambient pH conditions. Temporal trends are shown separately for fleshy macroalgal‐dominance, mixed macroalgal‐dominance and calcifying macroalgal‐dominance. Crosses (X) indicate the functional role(s) of each taxon while arrows indicate the overall direction of change in cover over the experimental period (↗ increase, ↘ decrease, = no clear change), while blank cells indicate absence of observations within a given community type or pH condition. Full species names are provided in Figure S5.

Key species	Taxonomic group	Calcifier	Primary producer	Filter‐feeder	Temporal change in cover								
					Extreme Low			Low			Ambient		
					Fleshy	Mixed	Calcified	Fleshy	Mixed	Calcified	Fleshy	Mixed	Calcified
*Bryopsis* sp., *A. stellata* , *F. petiolata*	Chlorophyta		X		↘	↘		↗	↗	↗	↗	↗	↗
*A. acetabulum*	Chlorophyta	X	X		↘	↘	↘	↘	↘	↘	↘	↘	↘
*Halopteris* sp.	Phaeophyceae		X		↘	↘	↘	↘	↘	↘	↘	↘	↘
*Dictyota* sp.	Phaeophyceae		X		↗	↗	↗	↗	↗	↗	=	↘	=
*P. pavonica*	Phaeophyceae	X	X		↘	↘		↘	↘		↘	↘	↘
*H. crouaniorum*	Rhodophyta		X		↗	↗	↗	=	↘		=	=	↘
*P. squamaria*	Rhodophyta		X		↘	↘	↘	↘	↘	↘	=	↘	↘
*H. virgatum*	Rhodophyta	X	X			↘		↘	↘	↘	↗	↗	↗
*H. farinosum, E* *. elongata*	Rhodophyta	X	X		↘	↘	↘	↘	↘	↘	↘	↘	↘
*Schizoporella* sp., *C. caminata*	Bryozoa	X		X	↘	↘	↘	↘	↘	↘	↗	↗	↗
*P. topsenti*	Porifera			X		↘	↘	↗	↗	↗	↘	↘	↘
*C. crambe*	Porifera			X	↘	↘	↘	↗	=	↘	↗	↘	↘
*C. clathrus*	Porifera	X		X		↘		↘			↗	↗	
*P. perforatus*	Crustacea	X		X	↘	↘	↘	=	=	↘	↗	↗	↗
*Ostrea* sp., *Lima lima*	Mollusca	X		X	↘		↘						
Serpulids	Polychaeta	X		X	↘	↘	↘	↗	↗	↗	↗	↗	↘
*C. dellechiajei, Didemnum* sp.	Tunicates			X	↘	↘	↘	↘	↘	↘	↘	↘	↘
Turf	Turf		X		↘	↘	↘	↗	↗	↗	=	=	=

**FIGURE 2:**
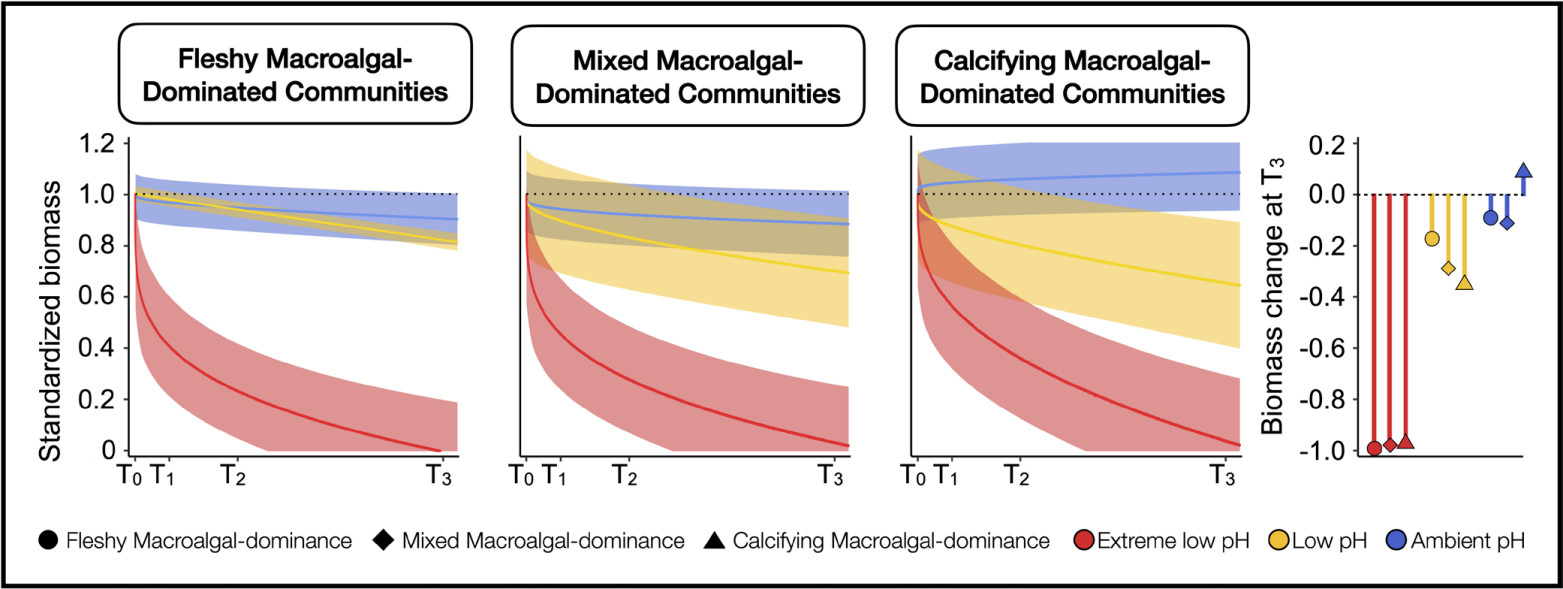
Biomass loss along the pH gradient over time. Predicted change in biomass across the three pH zones and assemblages over time. The solid lines represent the average modelled regression curves and the shaded areas indicate the 95% credible intervals of the predictions. Predicted change in biomass was standardized by T_0_. The lollipop plot represents the total change in biomass from the start (T_0_) to the end of the experiment (T_3_) across the three pH sites and the three assemblages.

All tiles exhibited a similar specific richness at T_0_ ranging from 20 ± 3 species for communities dominated by calcifying macroalgae to 22 ± 3 species for those dominated by fleshy macroalgae. Fleshy macroalgal‐dominated communities showed the largest changes, losing on average 7 ± 5, 7 ± 1 and 18 ± 4 species under ambient, low and extreme low pH conditions, respectively. Mixed macroalgal‐dominated communities experienced intermediate losses of 4 ± 2, 6.5 ± 5 and 17 ± 1 species across the same pH gradient. Calcifying macroalgal‐dominated communities were the least affected, with losses of 4 ± 4, 7 ± 2 and 14 ± 1 species at ambient, low and extreme low pH, respectively (see Figure S7). No new species colonized the tiles during the experiment and losses in richness directly reflect declines in existing taxa. Consequently, under low and extreme low pH, calcifying macroalgal‐dominated communities no longer retain their original composition, shifting instead toward more simplified assemblages dominated by a few low‐pH‐tolerant species (Table 2). Species loss was generally mirrored by proportional shifts in biomass, with responses ranging from slight gains up to 8% (credible intervals (CI) [−7%; 20%]) to decreases up to −12% (CI [−2%; −24%]) under ambient conditions. Under low and extreme low pH, biomass declined by −18% to −37% (CI [−21%; −15%] to [−58%; −8%]) and from −97% to −100% (CI [−100%; −71%] to [−100%; −80%]), respectively (Figure 2).

### 3.3 Ecosystem Functioning Under OA

**FIGURE 3:**
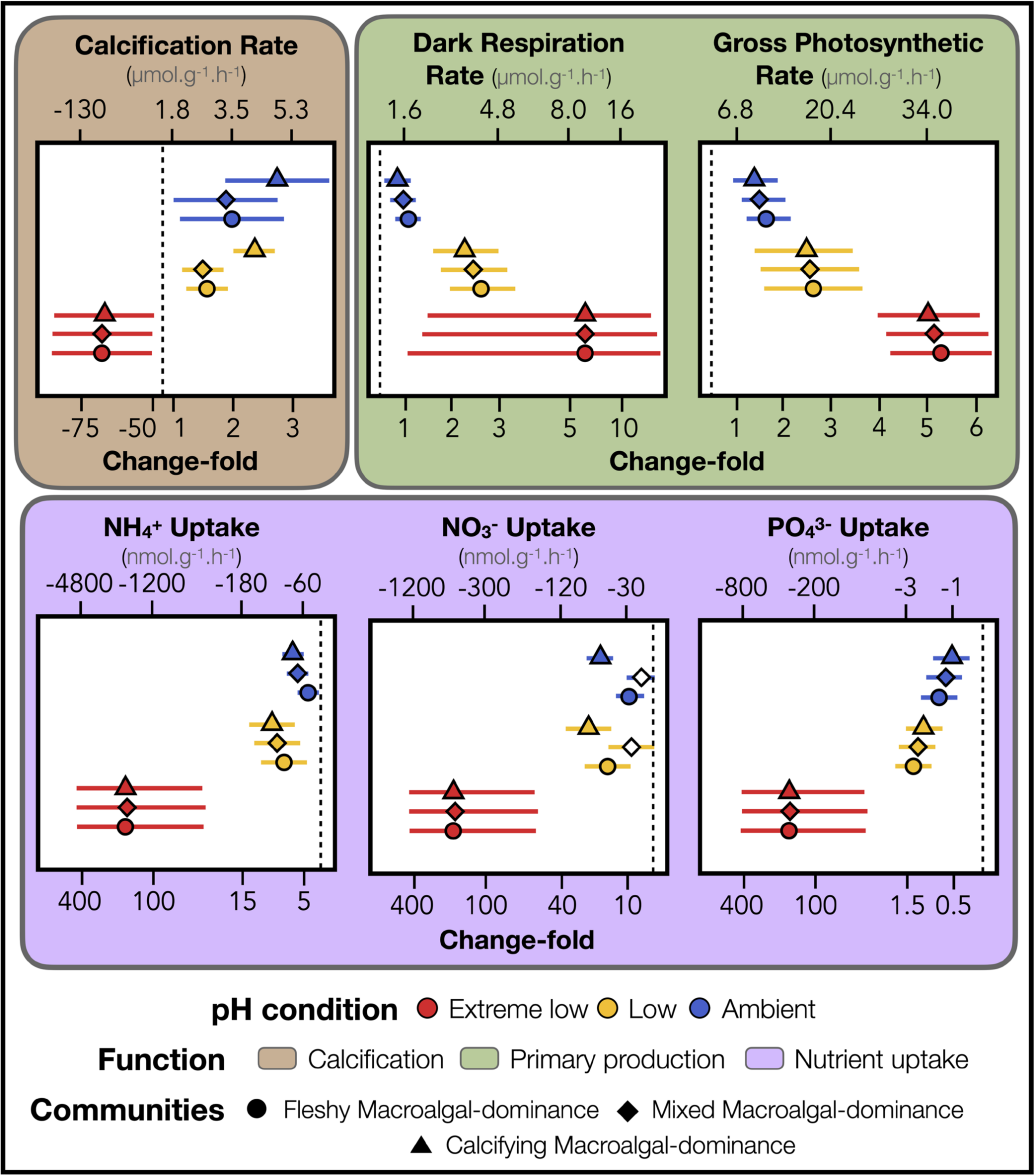
Ecosystem function responses to ocean acidification. Modelled changes over 100 days in six key functions across three pH conditions (extreme low, low and ambient pH), including calcification, dark respiration, gross photosynthesis, and nutrient (NH_4_^+^, NO_3_^−^ and PO_4_^3−^) uptake. These six processes were grouped into three core functions: calcification, primary production, and nutrient uptake. The ecosystem function responses were quantified based on the three dominant community types: fleshy macroalgal‐dominance (symbolled by triangles), mixed macroalgal‐dominance (symbolled by diamonds) and calcifying macroalgal‐dominance (symbolled by circles). The solid lines represent the corresponding credible intervals of the predictions. Symbol fill indicates whether credible intervals overlap zero, with filled symbols denoting responses whose credible intervals exclude zero.

Under ambient pH conditions, calcification rates increased over time, ranging from 3.5 to 5.1 μmol CaCO₃·g(DW)^−1^ h^−1^ (CI [−2.5; 9.5] to [−0.7; 11.1]) after 100 days (Figure 3). Under low pH conditions, calcification rates were lower, ranging from 3.0 to 4.6 μmol CaCO₃·g(DW)^−1^ h^−1^ (CI [0.8; 5.4] to [2.2; 6.8]). Under extreme low pH, calcification declined rapidly, with negative rates (i.e., net dissolution) recorded after only 7 days, corresponding to the observed loss of calcifying organisms. Gross photosynthetic rates increased over time for each pH condition, with the highest rates measured after 100 days under extreme low pH conditions (34.0–35.9 μmol O_2_.g(DW)^−1^ h^−1^, CI [26.7; 41.3] to [28.7; 43.2]). In comparison to the extreme treatment, gross photosynthetic rates were consistently lower under low pH (16.5–18.5 μmol O_2_.g(DW)^−1^ h^−1^, CI [8.7; 24.4] to [10.6; 26.4]) and ambient pH conditions (9.8–11.6 μmol O_2_ g(DW)^−1^ h^−1^, CI [6.8; 12.9] to [8.5; 14.6]). Similar results were observed for dark respiration rates, with higher rates under extreme low pH (from 11.2 to 12.0 μmol O_2_.g(DW)^−1^ h^−1^, CI [1.2; 21.2] to [2.0; 22.0]), consistently decreasing under low (from 3.6 to 4.5 μmol O_2_ g(DW)^−1^ h^−1^, CI [2.1; 5.1] to [3.0; 6.0]) and ambient pH conditions (from 0.8 to 1.7 μmol O_2_.g(DW)^−1^ h^−1^, CI [0.5; 1.2] to [1.4; 2.1]). Finally, nutrient uptake showed the strongest amplification across the pH gradient. Under ambient conditions, uptake rates remained low, ranging from 48.0 to 81.2 nmol g(DW)^−1^ h^−1^ for NH₄^+^ (CI [31.5; 64.4] to [64.9; 97.6]), from 8.7 to 76.6 for NO₃^−^ (CI [−9.0; 26.4] to [58.6; 94.6]), and from 0.2 to 0.4 for PO₄^3−^ (CI [0.1; 0.4] to [0.2; 0.5]). Uptake increased modestly under low pH, rising by factors of 1.3 to 8.4 for NH₄^+^ and PO₄^3−^ (CI [1.1; 1.6] to [4.1; 12.7]) and by 8.0 to 30.8 for NO₃^−^ (CI [−0.8; 16.9] to [17.6; 22.0]). Under extreme low pH, uptake rates increased dramatically by 199‐ to 254‐fold relative to ambient conditions (CI [14.0; 385.6] to [86.0; 421.9]).

**FIGURE 4:**
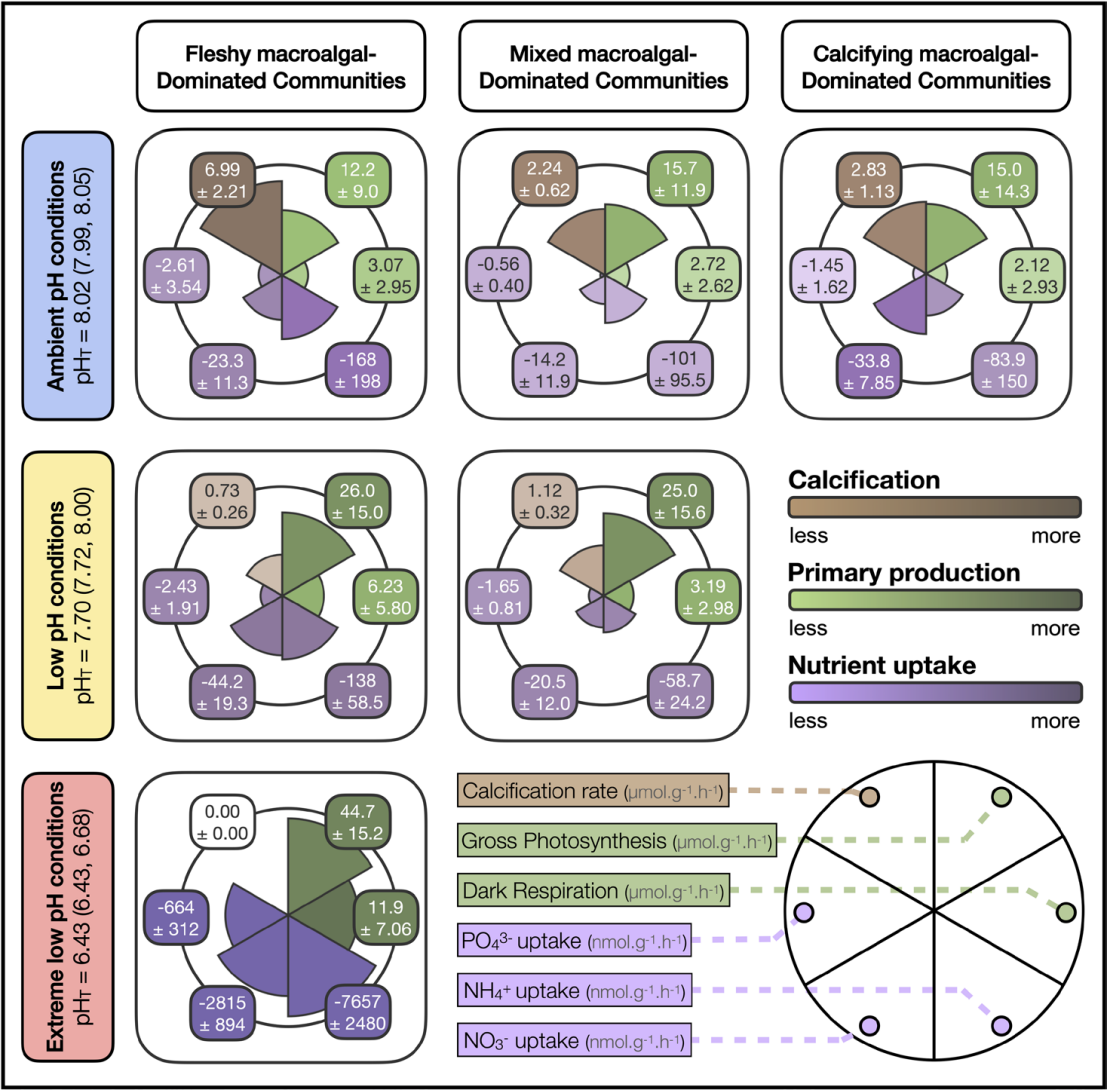
Long‐term ecosystem functioning responses to ocean acidification on temperate benthic communities. Rates of six key processes across the three dominant community types (i.e., fleshy macroalgal‐dominance, mixed macroalgal‐dominance and calcifying macroalgal‐dominance) and the three pH conditions (extreme low, low and ambient) including mean pH_T_ values followed by the 25th and 75th percentiles into parenthesis. The six processes (i.e., calcification, dark respiration and gross photosynthetic rates and nutrient [NH_4_^+^, NO_3_^−^ and PO_4_^3−^] uptake were grouped into three core functions as calcification [brown], primary production [green] and nutrient cycling [purple]). Calcifying macroalgal‐dominated communities were absent in extreme low and low pH conditions, and mixed macroalgal‐dominated communities were absent in extreme low pH conditions. The size and colour of the bars in the plot indicate the absolute values. All values are displayed with the average estimate ± standard error from bayesian models.

To confirm that our results from the short‐term experiment are representative of longer time frames, we quantified EF on tiles exposed to the three pH conditions for nearly 15 years (Figure 4). We found that calcifying macroalgae were never dominant under low pH and were absent in extreme low pH conditions. Consistent with our findings from analysis of the transplanted tiles, calcification rates were significantly higher under ambient pH conditions, ranging from 2.2 to 7.0 μmol CaCO_3_ g(DW)^−1^ h^−1^ (CI [1.6; 2.9] to [4.8; 9.2]), than under low pH, where rates were reduced to between 0.7 and 1.1 μmol CaCO_3_ g(DW)^−1^ h^−1^ (CI [0.5; 1.0] to [0.8; 1.4]). No calcification was observed under extreme low pH. Dark respiration and gross photosynthetic rates showed a consistent trend, with the highest values under extreme low pH conditions, reaching 11.9 (CI [4.8; 19.0]) and 44.7 μmol O_2_.g(DW)^−1^ h^−1^ (CI [29.5; 59.9]) and decreasing to values up to 2.1 (CI [−0.8; 5.0]) and 12.2 μmol O_2_.g(DW)^−1^ h^−1^ (CI [3.2; 21.2]) under ambient pH conditions, respectively. Nutrient uptake rates were comparable to those observed in transplant experiments, although slightly higher, with greater uptake rates under extreme low pH. NH_4_^+^ uptake reached 7.7 μmol g(DW)^−1^ h^−1^ (CI [5.2; 10.1]), NO_3_^−^ uptake reached 2.8 μmol g(DW)^−1^ h^−1^ (CI [1.9; 3.7]) and PO₄^3−^ uptake reached 0.7 μmol g(DW)^−1^ h^−1^ (CI [0.4; 1.0]) with rates decreasing as pH increased.

## 4 Discussion

By examining changes in benthic communities using settlement tiles deployed along a natural CO_2_ gradient, our study reveals consistent shifts in community structure and EF under increasing OA. Species richness and biomass declined with decreasing pH. These changes in community structure were accompanied by shifts in EF, as calcification rates in communities experiencing long‐term exposure to acidified conditions declined by approximately 50%–90% under low pH and were entirely absent under extreme low pH, consistent with the disappearance of calcifying organisms. In contrast, fleshy macroalgal species thrived under increased CO_2_ levels, accompanied by higher rates of gross photosynthesis, which increased up to twofold under low pH and up to fourfold under extreme low pH, as well as enhanced nutrient uptake, rising by approximately 110%–180% under low pH and by up to ~250‐fold under extreme low pH relative to ambient conditions. Together, these results demonstrate a predictable shift in community dominance from calcifying to macroalgal taxa, accompanied by reduced diversity, declining calcification, and increasing primary production and nutrient turnover under elevated CO_2_.

### 4.1 Biodiversity Loss and Ecosystem Simplification

Our results show that benthic communities exposed to increasing OA diverged clearly into ecological winners and losers (Baskin 1998) across a pH gradient. Similar to previous studies (Hu et al. 2024), we observed that highly adaptable species, particularly fleshy macroalgae and turf‐forming taxa, increased in dominance under acidified conditions, while calcifying organisms declined sharply along the gradient (Cornwall et al. 2017). The proliferation of fleshy macroalgae was associated with a reduction in community structural complexity, as low‐profile, fast‐growing taxa increasingly dominated settlement tiles under low and extreme low pH (Agostini et al. 2021). Such dominance likely contributes to reinforcing community‐level shifts by limiting space and resources available for slower‐growing or structurally complex taxa (Milazzo et al. 2019). Consequently, benthic communities became increasingly simplified, with a measurable decline in species richness, consistent with patterns of biotic homogenization under environmental stress (Clavel et al. 2011). However, our results showcase that biodiversity loss did not translate uniformly into functional decline. Indeed, some ecosystem functions were amplified, indicating that functional responses did not track structural changes uniformly, as previously reported in marine systems such as tropical coral reefs (Carlot et al. 2022) or temperate rocky reefs (Teixido et al. 2024).

### 4.2 Ecosystem Functioning Shifts

The observed increase in photosynthetic rates due to OA has been previously highlighted for phytoplankton (Mackey et al. 2015) but remains controversial for macroalgal‐dominated benthic communities due to the high variability in responses among macroalgal species (Wada et al. 2025; Cornwall et al. 2024; Paine et al. 2023). In this study, we find that macroalgal‐dominated ecosystems might be enhanced by OA and that nutrient availability, such as nitrogen (in the form of NH_4_^+^ and NO_3_^−^) and phosphorus (in the form of PO_4_^3−^) may play a key role in supporting higher photosynthetic rates under OA (Helliwell 2023), with rates proportionally rising as nutrient concentrations increase (Roleda and Hurd 2019). Although phosphorus is expected to remain less available than nitrogen in marine systems due to global change (Penuelas et al. 2020), we show that benthic communities may enhance uptake of both nutrients to sustain high photosynthetic activity. In contrast, calcification declines with increasing OA and disappears entirely under extreme conditions as expected (Cornwall et al. 2022). This shift toward non‐calcifying, fast‐growing taxa may reshape carbon cycling by reducing carbonate deposition while maintaining or increasing organic matter production and nutrient turnover (Romanó de Orte et al. 2021). Our results indicate that the replacement of calcifying taxa by fleshy macroalgae drives a functional transition from calcification‐dominated processes toward higher primary production and nutrient fluxes. Even with a significant decrease in macroalgal biomass, we demonstrate that OA can reallocate ecosystem‐level energy and matter flows, potentially influencing carbon cycling, organic matter production and export, and trophic transfer in coastal benthic systems (Ullah et al. 2018; Wada et al. 2021). Overall, our findings suggest that elevated CO_2_ levels could stimulate and promote shifts to marine ecosystems dominated by macroalgal communities (Goldenberg et al. 2017).

### 4.3 Long‐Term Consequences in a Changing World

Here, we highlight that apparent increases in photosynthesis and nutrient uptake primarily reflect the strong reduction in total community biomass along the pH gradient, as OA favours low‐complexity, low‐biomass taxa such as *Dictyota* sp., *H. crouaniorum* and turf‐forming algae (Teixido et al. 2024). These taxa are characterized by rapid lateral expansion and efficient space occupation, and are therefore less constrained by surface availability than structurally complex organisms such as calcifying algae or corals (Steneck and Dethier 1994). Because our study was conducted on settlement tiles with a fixed and limited surface area, biomass standardization provides the most appropriate metric to compare functional rates across contrasting community states. When EF is instead standardized by surface area (see Figure S8), photosynthetic and overall nutrient uptake rates remain slightly higher under low pH compared to ambient conditions but decline sharply under extreme low pH, indicating that total biomass remains a key driver of EF at the community level (Lohbeck et al. 2015).

In the frame of our study, communities were not exposed to additional pressures, allowing us to isolate the effects of OA on EF. However, Mediterranean marine ecosystems have been rarely subjected to a single pressure over the past decades (Carlot et al. 2025). Ocean warming, deoxygenation, altered stratification, reduced mixed‐layer depth and shifts in nutrient supply are expected to interact with OA to shape ecosystem processes. For example, warming‐induced stratification can reduce vertical nutrient supply, constraining primary productivity and altering competitive dynamics among primary producers (Boyd et al. 2015; Riebesell et al. 2008). In this context, the conjunction of multiple pressures over time would likely modify EF rates primarily when expressed per unit area with likely higher EF rates under ambient conditions. For instance, although *Dictyota* sp. was among the dominant species found under extreme low pH in our study, its thermal sensitivity (> 30°C; Kaschner et al. 2016) suggests that the combined effects of OA and warming would further erode species diversity and biomass, amplifying declines in area‐standardized EF and underscoring uncertainty about EF and resilience following biodiversity loss in the long term (Oliver et al. 2015).

## 5 Conclusion

Our study demonstrates that OA drives a predictable reorganization of temperate benthic communities, characterized by a shift from calcifying macroalgal assemblages to low‐complexity, fast‐growing taxa such as *Dictyota* sp. and turf‐forming algae. This compositional transition is driven by the loss of calcifying species and occurs without evidence of species replacement, resulting in simplified communities dominated by a small number of low‐pH‐tolerant taxa. These structural changes translate into clear functional shifts, as community calcification declines sharply and disappears under extreme low pH, whereas gross photosynthesis and nutrient uptake increase, particularly when expressed per unit biomass. Our results indicate that total community biomass, rather than surface area, is the primary determinant of EF under OA in constrained habitats such as settlement tiles. Although functioning per unit area would likely decline under extreme conditions or in the presence of additional pressures, biomass‐standardized metrics reveal that remaining communities can maintain or even amplify specific functions. Together, these findings show that OA reshapes EF in Mediterranean macroalgal communities through functional transitions driven by community reorganization, rather than uniform declines across processes.

## Author Contributions

All authors have agreed to the submission of this manuscript and take responsibility for the integrity, accuracy and ethics of the work. J. Carlot and N. Teixidó are responsible for the overall integrity of the manuscript. All authors made substantial intellectual contributions and meet the authorship criteria of *Ecology Letters*. Conceptualization: J. Carlot, S. Comeau, J.‐P. Gattuso and N. Teixidó. Data curation: J. Carlot, A. Chiarore, A. Mirasole, S. Alliouane and N. Teixidó. Formal analysis: J. Carlot. Investigation: J. Carlot, A. Chiarore, A. Mirasole and N. Teixidó. Writing (first draft) J. Carlot. Writing (review and editing) J. Carlot, S. Comeau, A. Chiarore, A. Mirasole, S. Alliouane, C.L. Hurd, F. Micheli, J.‐P. Gattuso and N. Teixidó. Funding: J. Carlot and N. Teixidó.

## Funding

This work was supported by the French National Research Agency Investments for the Future, ANR‐17‐MOPGA‐0001. National Recovery and Resilience Plan (NRRP), Mission 4 Component 2 Investment 1.4, CN_00000033. Direcció General de Recerca, Generalitat de Catalunya, 2024 BP 00106.

## Conflicts of Interest

The authors declare no conflicts of interest.

## Supporting information


**Table S1:** Measured and estimated seawater physicochemical parameters at T0 of each incubation of the transplant experiment.
**Table S2:** Measured and estimated seawater physicochemical parameters at T0 of each incubation of the historic tiles experiment.
**Figure S1:** Experimental design and setup.
**Figure S2:** Evolution of the transplanted tiles over time.
**Figure S3**. Raw ecosystem function rates in response to ocean acidification.
**Figure S4:** Photo‐irradiance curves under the three pH conditions.
**Figure S5:** Changes in species cover along the pH gradient.
**Figure S6**. Differences in initial community composition among communities.
**Figure S7:** Species richness change along the pH gradient over time.
**Figure S8:** Surface‐area‐standardized ecosystem functions to acidification.

## Data Availability

The data and code to generate all figures are available on Zenodo (https://doi.org/10.5281/zenodo.18653612) and GitHub (https://github.com/JayCrlt/BenthFun).
