# Supplementary material for: Unravelling Marine Benthic Functioning Shifts Under Ocean Acidification
Source: Ecol Lett. 2026 Apr 3;29(4):e70376. doi: 10.1111/ele.70376 (PMC13049105; doi:10.1111/ele.70376)
Supplement: Supplementary file 1 — Table S1: Measured and estimated seawater physicochemical parameters at T0 of each incubation of the transplant experiment. Table S2: Measured and estimated seawater physicochemical parameters at T0 of each incubation of the historic tiles experiment. Figure S1: Experimental design and setup. Figure S2: Evolution of the transplanted tiles over time. Figure S3. Raw ecosystem function rates in response to ocean acidification. Figure S4: Photo‐irradiance curves under the three pH conditions. Figure S5: Changes in species cover along the pH gradient. Figure S6. Differences in initial community composition among communities. Figure S7: Species richness change along the pH gradient over time. Figure S8: Surface‐area‐standardized ecosystem functions to acidification. [file ELE-29-0-s001.pdf]

Supplementary information of Carlot *et al*. (2026) Unravelling marine benthic functioning shifts under ocean acidification

List of supplementary tables

Supplementary table 1 | Measured and estimated seawater physicochemical parameters at T_0 of each incubation of the transplant experiment.
Supplementary table 2 | Measured and estimated seawater physicochemical parameters at T_0 of each incubation of the historic tiles experiment.

List of supplementary figures

Supplementary figure 1 | Experimental design and setup
Supplementary figure 2 | Evolution of the transplanted tiles over time
Supplementary figure 3 | Raw ecosystem function rates in response to ocean acidification
Supplementary figure 4 | Photo-irradiance curves under the three pH conditions
Supplementary figure 5 | Changes in species cover along the pH gradient
Supplementary figure 6 | Differences in initial community composition among communities
Supplementary figure 7 | Species richness change along the pH gradient over time
Supplementary figure 8 | Surface-area-standardized ecosystem functions to acidification

Data and code availability

All methods are described within the paper. The data to generate all figures is available at https://github.com/JayCrlt/BenthFun

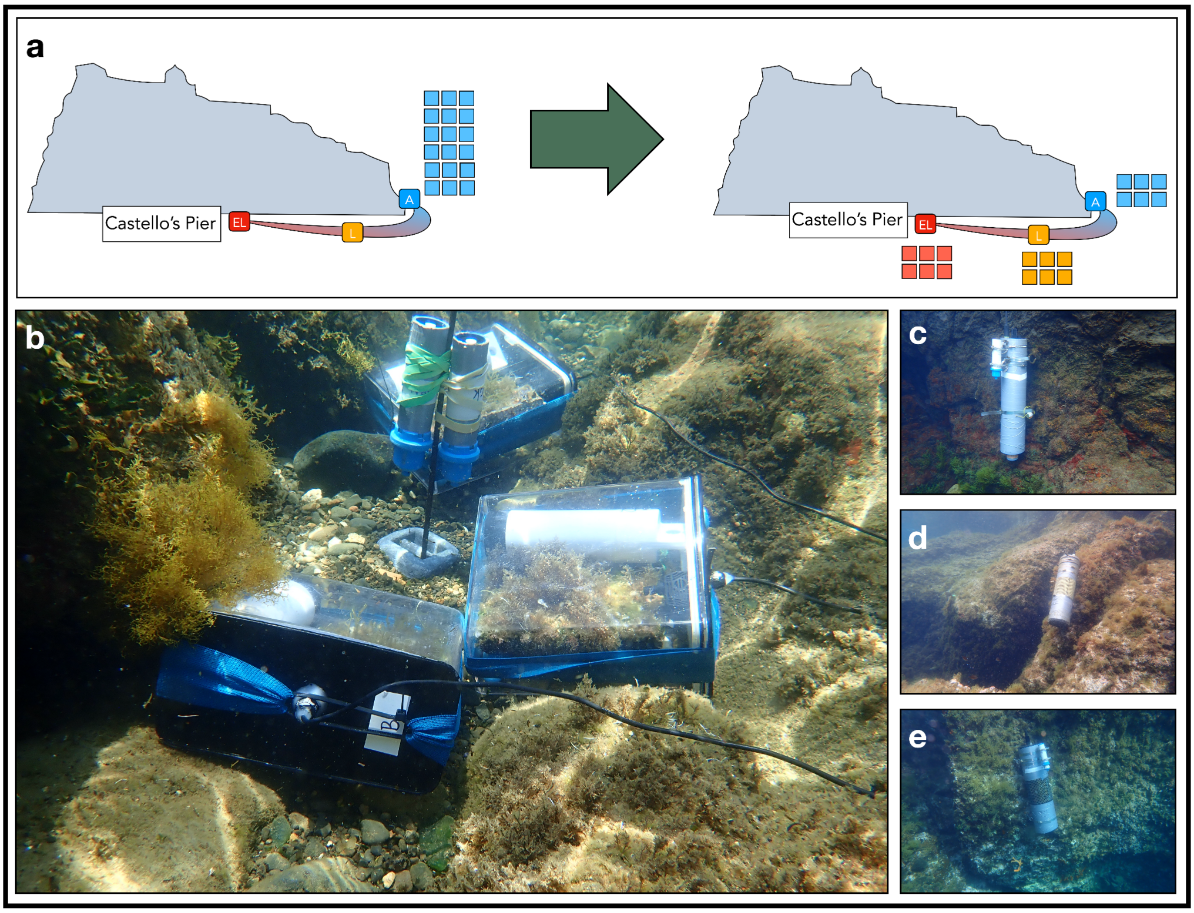

Supplementary figure 1 | Experimental design and setup. **a.** Experimental design of the transplanted tile experiment. Each blue square represents a tile initially placed in ambient pH conditions (left) before being moved to: extreme low pH conditions (n = 6 tiles, EL, red), low pH conditions (n = 6 tiles, L, yellow), or remaining in ambient pH conditions as a control (n = 6 tiles, A, blue). **b.** Incubation chambers used during one-hour incubations were equipped with O_2 sensors. Two PAR (Photosynthetically Active Radiation) sensors were deployed during the experiment. **c.** SeaFET™ Ocean pH sensor (Satlantic) deployed in extreme low pH conditions and **d, e.** SAMI-pH sensors (Sunburst) deployed in low and ambient pH conditions, along the transplanted tiles at a depth between 0.5 and 3m.

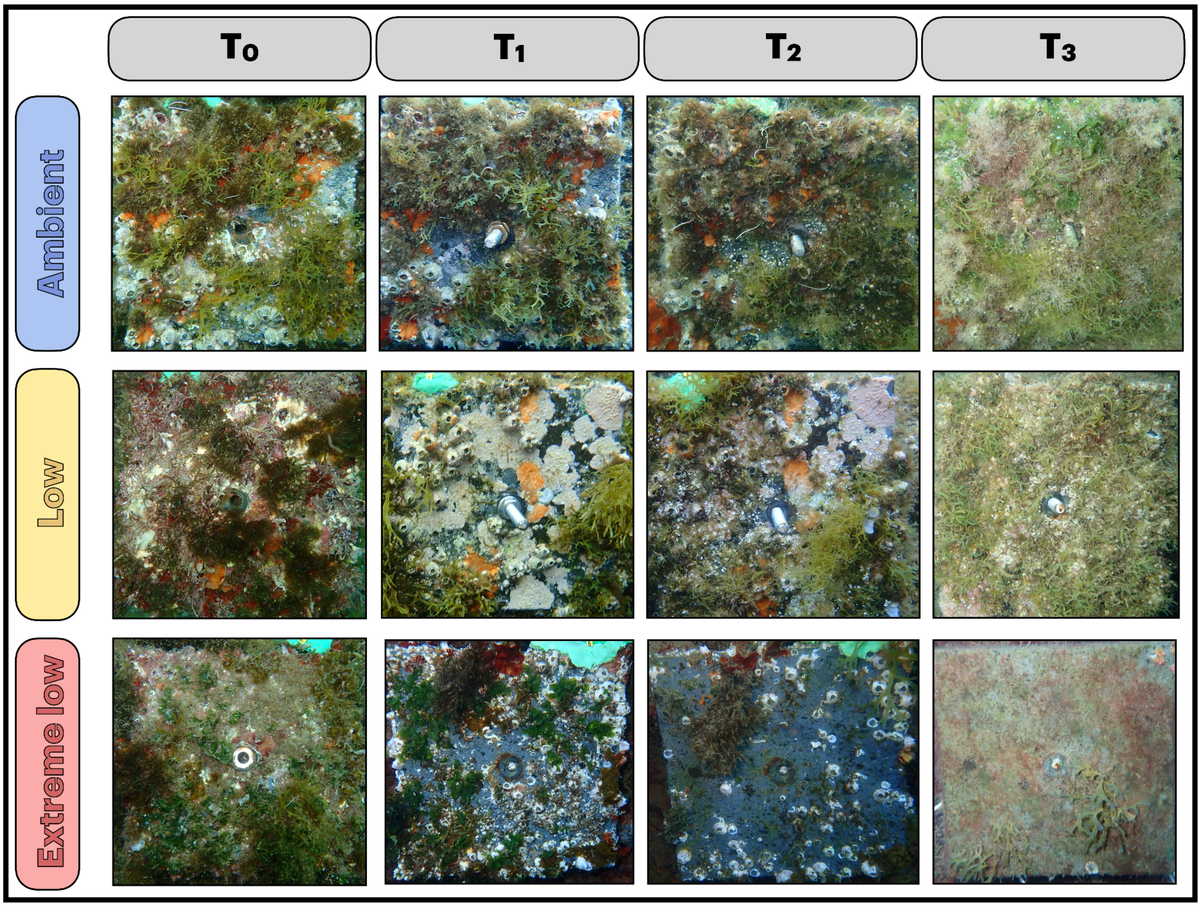

Supplementary figure 2 | Temporal dynamics of the transplanted tiles over time. Each row represents a single transplanted tile across four time points: T_0 (before transplant), T_1 (7 days post-transplant), T_2 (30 days post-transplant), and T_3 (120 days post-transplant). Each row is organized by pH conditions, with ambient pH at the top, low pH in the middle, and extreme low pH at the bottom.

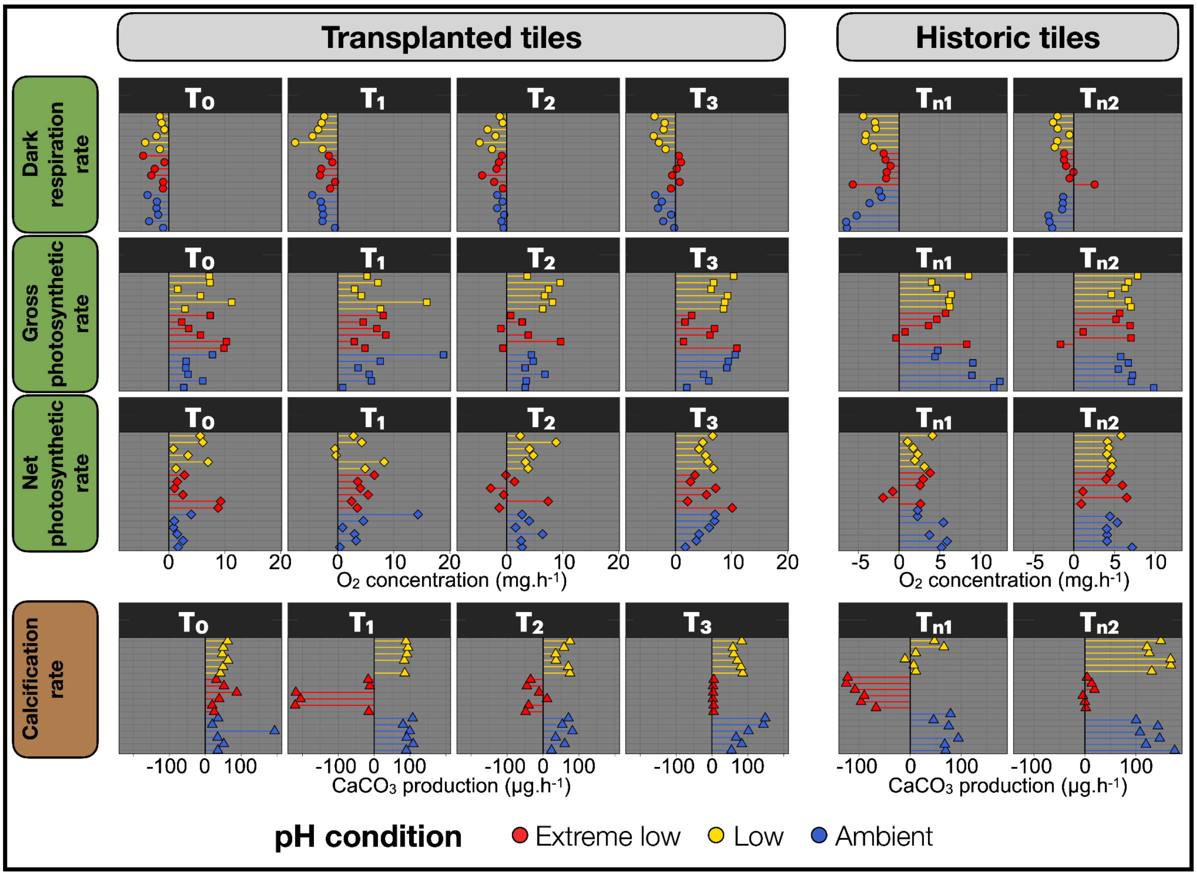

Supplementary figure 3 | Raw ecosystem function rates in response to ocean acidification over time. Each row represents different ecosystem functions: dark respiration rate (mg O_2 h^{-1} per tile), gross photosynthetic rate mg O_2 h^{-1} per tile) net photosynthetic rate (mg O_2 h^{-1} per tile) and calcification rate (µg $CaCO_3$ h^{-1} per tile). For each pH condition, six tiles were monitored, with extreme low pH conditions in red, low pH conditions in yellow, and ambient pH conditions in blue.

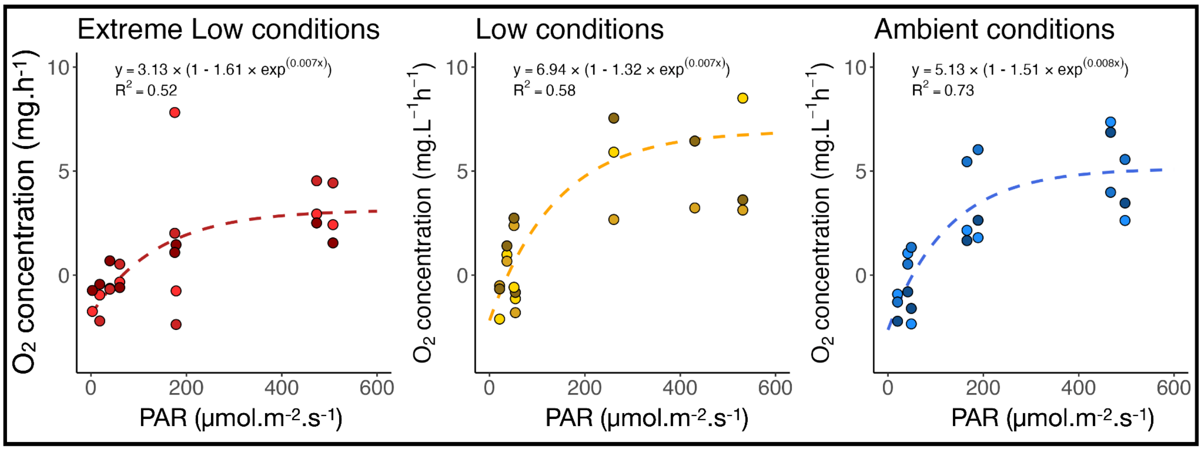

Supplementary figure 4 | Photo-irradiance curves under the three pH conditions. Each scatter plot shows the change in O_2 concentration ($mg.h^{-1}$) as a function of PAR ($\mu mol.m^{-2}.s^{-1}$). Three tiles were measured to capture the dynamics of maximum photosynthetic activity for each pH condition (n= 7 to 8 incubations per tile over a day from 06:00 am to 08:00 pm). The data were fitted using the equation $y = \alpha(1 - \beta e^{\gamma x})$, yielding R^2 values of 0.52 (extreme low pH), 0.58 (low pH), and 0.73 (ambient pH), where α represents the maximum photosynthetic rate, and β and γ are constants modifying the curvature and saturation rate. Each colored dot represents a different tile.

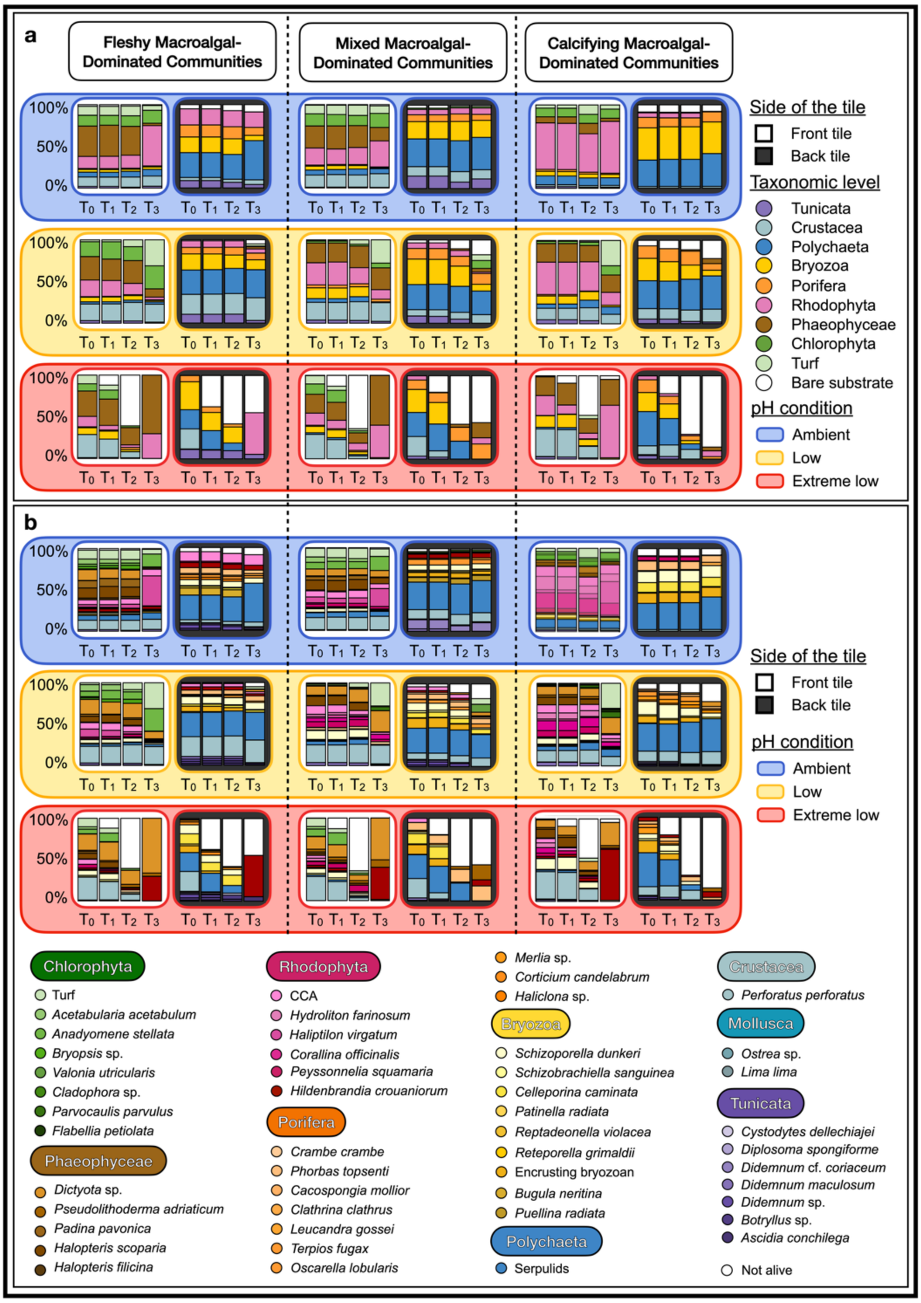

Supplementary figure 5 | Biomass loss and changes in benthic cover along a natural pH gradient through time. Bars represent mean benthic cover on the front (white background) and back (black background) surfaces of settlement tiles across three pH conditions (ambient, low, and extreme low). Tiles were classified as fleshy macroalgal-dominated, mixed macroalgal-dominated, or calcifying macroalgal-dominated communities (n = 6 per community type) based on taxonomy, growth form, and the presence of calcareous structures. Community composition was assessed at four time points: before transplantation (T_0), and 7 (T_1), 30 (T_2), and 120 (T_3) days after transplantation. **a.** Changes in benthic cover summarized at the taxonomic group level (Turf, Chlorophyta, Phaeophyceae, Rhodophyta, Porifera, Bryozoa, Polychaeta, Crustacea, Tunicata, and bare substrate). **b.** Changes in benthic cover resolved at the species level (49 taxa), grouped into the same major taxonomic categories and bare substrate.

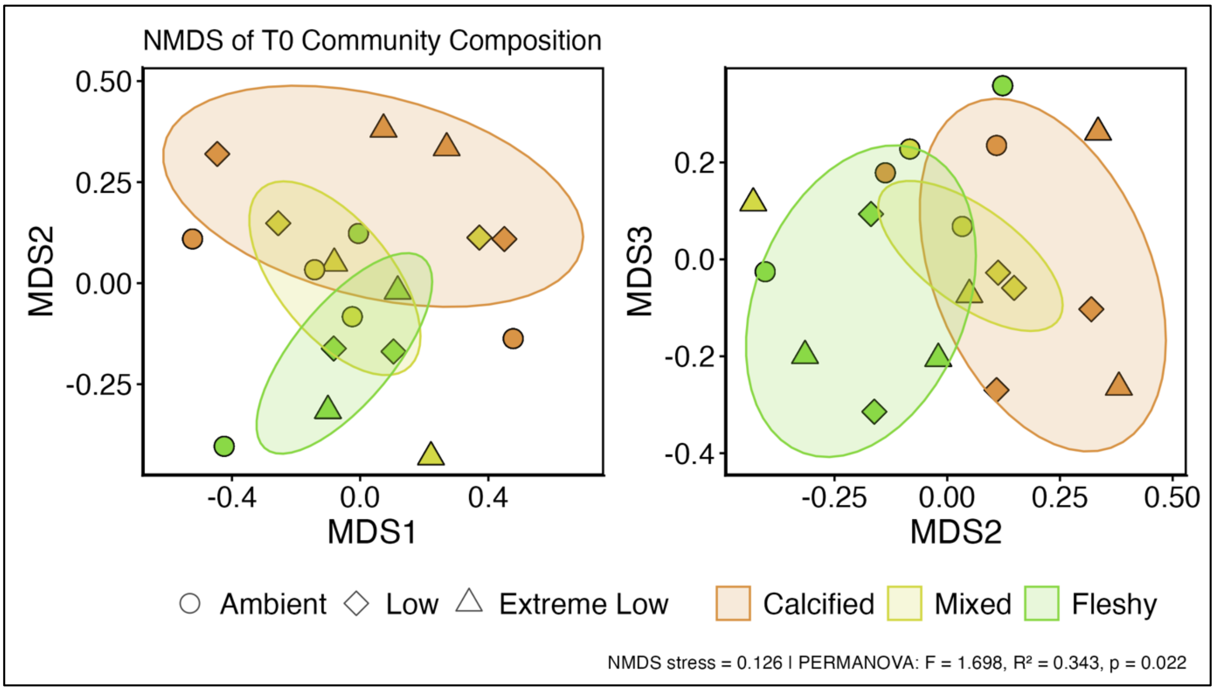

Supplementary figure 6 | Differences in initial community composition among assemblage types at T_0. Non-metric multidimensional scaling (nMDS) ordinations of benthic community composition at the start of the experiment (T_0), based on species-level cover data. Panels show ordinations using different axis combinations (MDS1–MDS2 and MDS2–MDS3). Points represent individual tiles, coloured by community type (calcifying, mixed, and fleshy macroalgal-dominated communities) and shaped by pH treatment (ambient, low, and extreme low) where they will be transplanted. Ellipses represent 95% confidence intervals around group centroids, illustrating significant differences in species composition among community types (PERMANOVA, $p < 0.05$).

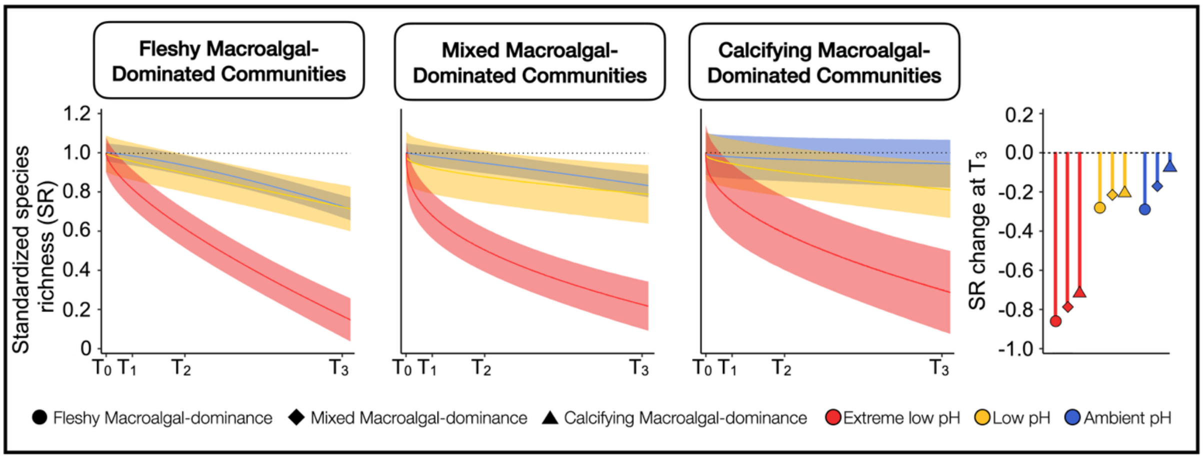

Supplementary figure 7 | Species richness change along the pH gradient over time. Predicted change in species richness across the three pH zones and community assemblages over time. Solid lines represent the mean modeled regression curves, and shaded areas indicate the 95% credible intervals of the predictions. Changes in richness were standardized relative to initial conditions (T_0).

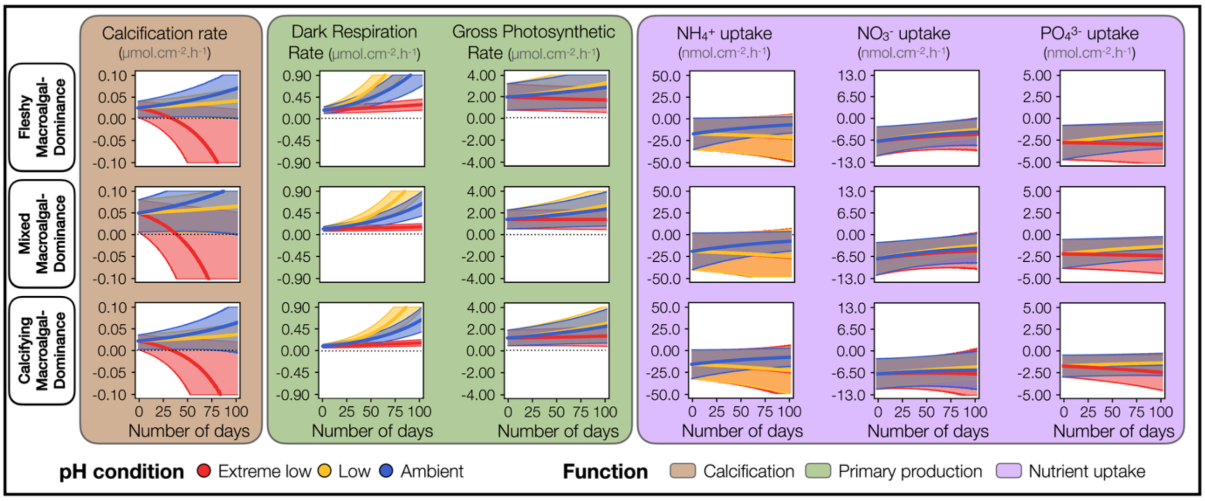

Supplementary figure 8 | Surface-area-standardized ecosystem function responses to ocean acidification over time. Modeled changes over time in six key functions including calcification, dark respiration, gross photosynthesis, and nutrient (NH_4^+, NO_3^-, and PO_4^{3-}) uptake across three pH conditions (extreme low, low, and ambient pH). These four processes were grouped into three core functions such as calcification, primary production, and nutrient cycling. The ecosystem function responses were quantified based on the three dominant community types: fleshy algae-dominance, mixed algae-dominance, and calcifying algae-dominance. The solid lines represent the average modeled regression curves and the shaded areas indicate the corresponding CIs of the predictions

Supplementary table 1 | Measured and estimated seawater physiochemical parameters at T_0 of each incubation of the transplant experiment. The following parameters below are NH_4^+ concentration, NO_3^- concentration, PO_4^- concentration, temperature (T), total alkalinity (A_T), dissolved inorganic carbon (C_T), pH_T, pCO_2, calcite (Ωc) and aragonite (Ωa) saturation. Values are means ± SD. Calculated concentrations of C_T, pCO_2, Ωc, and Ωa were defined using the seacarb package in R.

Date	Stage	pH Conditions	NH_4^+ (mmol.m^{-3})	NO_3^- (mmol.m^{-3})	PO_4^{3-} (mmol.m^{-3})	T (°C)	A_T (µmol kg^{-1})	C_T (µmol kg^{-1})	pH_T	pCO_2 (µatm)	Ωc	Ωa
08/05/2023	T_0	Extreme Low	0,81 ± 0,15	0,20 ± 0,16	0,04 ± 0,00	19,08 ± 0,01	2612,66 ± 7,49	4045,60 ± 167,16	6,16 ± 0,05	43358,93 ± 5234,86	0,08 ± 0,01	0,05 ± 0,01
09/05/2023	T_0	Low	1,37 ± 0,49	0,66 ± 0,17	0,16 ± 0,01	19,05 ± 0,22	2656,92 ± 1,66	2485,89 ± 177,70	7,81 ± 0,38	1072,77 ± 910,97	3,52 ± 2,48	2,28 ± 1,61
10/05/2023	T_0	Ambient	1,21 ± 0,58	0,45 ± 0,50	0,15 ± 0,12	19,04 ± 0,30	2662,40 ± 17,39	2352,09 ± 4,99	8,10 ± 0,03	401,24 ± 29,38	5,51 ± 0,38	3,57 ± 0,25
22/05/2023	T_1	Extreme Low	3,95 ± 0,69	0,48 ± 0,39	0,17 ± 0,11	18,46 ± 0,01	2566,13 ± 16,01	3839,23 ± 271,62	6,21 ± 0,08	37878,79 ± 7554,41	0,09 ± 0,02	0,06 ± 0,01
23/05/2023	T_1	Low	2,67 ± 0,75	0,26 ± 0,08	0,06 ± 0,02	19,02 ± 0,33	2538,39 ± 3,68	2378,95 ± 145,08	7,80 ± 0,33	983,69 ± 746,62	3,22 ± 1,96	2,09 ± 1,27
25/05/2023	T_1	Ambient	2,38 ± 0,07	0,44 ± 0,08	0,08 ± 0,03	20,10 ± 0,31	2548,56 ± 8,41	2261,84 ± 5,99	8,06 ± 0,00	426,39 ± 3,48	5,06 ± 0,05	3,29 ± 0,03
19/06/2023	T_2	Extreme Low	3,37 ± 1,49	0,21 ± 0,11	0,18 ± 0,06	24,48 ± 0,08	2707,23 ± 2,09	2987,45 ± 361,63	6,91 ± 0,52	11236,71 ± 11082,38	0,74 ± 0,71	0,49 ± 0,47
20/06/2023	T_2	Low	2,69 ± 0,16	0,19 ± 0,02	0,08 ± 0,01	24,95 ± 0,11	2677,63 ± 1,08	2369,84 ± 61,69	8,00 ± 0,10	534,31 ± 147,51	5,59 ± 1,01	3,68 ± 0,66
21/06/2023	T_2	Ambient	2,58 ± 0,65	0,30 ± 0,02	0,15 ± 0,03	25,01 ± 0,24	2679,83 ± 6,36	2357,32 ± 7,01	8,03 ± 0,02	491,24 ± 21,06	5,83 ± 0,23	3,84 ± 0,15
12/09/2023	T_3	Extreme Low	0,94 ± 0,02	0,52 ± 0,41	0,13 ± 0,10	24,13 ± 0,01	2577,67 ± 22,30	3380,53 ± 704,39	6,46 ± 0,44	28194,96 ± 24681,02	0,23 ± 0,20	0,15 ± 0,13
13/09/2023	T_3	Low	2,27 ± 1,20	0,87 ± 0,02	0,10 ± 0,02	24,87 ± 0,18	2607,40 ± 7,17	2393,80 ± 17,86	7,85 ± 0,02	772,00 ± 51,58	4,05 ± 0,15	2,67 ± 0,10
14/09/2023	T_3	Ambient	1,63 ± 0,10	0,38 ± 0,19	0,20 ± 0,19	24,86 ± 0,04	2587,47 ± 10,00	2282,66 ± 9,67	8,01 ± 0,00	490,92 ± 2,39	5,46 ± 0,01	3,60 ± 0,01

Supplementary table 2 | Measured and estimated seawater physiochemical parameters at T_0 of each incubation of the historic tiles experiment. The following parameters below are NH_4^+ concentration, NO_3^- concentration, PO_4^- concentration, temperature (T), total alkalinity (A_T), dissolved inorganic carbon (C_T), pH_T, pCO_2, calcite (Ωc) and aragonite (Ωa) saturation. Values are means ± SD. Calculated concentrations of C_T, pCO_2, Ωc, and Ωa were defined using the seacarb package in R.

Date	Stage	pH Conditions	NH_4^+ (mmol.m^{-3})	NO_3^- (mmol.m^{-3})	PO_4^{3-} (mmol.m^{-3})	T (°C)	A_T (µmol kg^{-1})	C_T (µmol kg^{-1})	pH_T	pCO_2 (µatm)	Ωc	Ωa
13/06/2023	T_{n1}	Extreme Low	4,80 ± 0,16	0,83 ± 0,39	0,20 ± 0,12	24,63 ± 0,03	2790,01 ± 1,79	2940,96 ± 1,16	7,00 ± 0,01	6683,69 ± 90,23	0,71 ± 0,01	0,46 ± 0,01
14/06/2023	T_{n1}	Low	2,47 ± 1,24	0,48 ± 0,24	0,09 ± 0,00	24,59 ± 0,11	2692,34 ± 30,48	2477,18 ± 142,29	7,84 ± 0,22	891,99 ± 491,60	4,17 ± 1,68	2,75 ± 1,10
15/06/2023	T_{n1}	Ambient	2,74 ± 1,64	0,51 ± 0,17	0,06 ± 0,04	23,13 ± 0,13	2638,94 ± 12,51	2332,17 ± 21,10	8,04 ± 0,01	472,61 ± 20,18	5,50 ± 0,13	3,61 ± 0,09
19/09/2023	T_{n2}	Extreme Low	2,23 ± 0,51	0,93 ± 0,10	0,07 ± 0,00	24,79 ± 0,05	2641,43 ± 38,86	3679,80 ± 396,00	6,26 ± 0,14	36716,1 ± 12417,8	0,13 ± 0,04	0,08 ± 0,03
18/09/2023	T_{n1}	Low	2,47 ± 0,53	0,70 ± 0,14	0,03 ± 0,01	25,12 ± 0,13	2531,98 ± 14,13	2259,22 ± 6,85	7,96 ± 0,01	550,86 ± 15,73	4,91 ± 0,12	3,24 ± 0,08
15/09/2023	T_{n2}	Ambient	1,73 ± 0,15	0,83 ± 0,02	0,09 ± 0,03	24,86 ± 0,17	2591,91 ± 16,57	2279,32 ± 13,64	8,03 ± 0,00	475,90 ± 3,23	5,59 ± 0,06	3,68 ± 0,04
